# Two dimensional (2D) reduced graphene oxide (RGO)/hexagonal boron nitride (*h*-BN) based nanocomposites as anodes for high temperature rechargeable lithium-ion batteries

**DOI:** 10.1038/s41598-020-58439-z

**Published:** 2020-02-05

**Authors:** Yasmin Mussa, Faheem Ahmed, Muhammad Arsalan, Edreese Alsharaeh

**Affiliations:** 1 College of Science and General Studies, Alfaisal University, P. O. Box 50927, Riyadh, 11533 Saudi Arabia; 2 EXPEC Advanced Research Center, Saudi Aramco, P. O. Box 5000, Dhahran, 31311 Saudi Arabia

**Keywords:** Batteries, Two-dimensional materials

## Abstract

With lithium-ion (li-ion) batteries as energy storage devices, operational safety from thermal runaway remains a major obstacle especially for applications in harsh environments such as in the oil industry. In this approach, a facile method via microwave irradiation technique (MWI) was followed to prepare Co_3_O_4_/reduced graphene oxide (RGO)/hexagonal boron nitride (*h*-BN) nanocomposites as anodes for high temperature li-ion batteries. Results showed that the addition of *h*-BN not only enhanced the thermal stability of Co_3_O_4_/RGO nanocomposites but also enhanced the specific surface area. Co_3_O_4_/RGO/*h*-BN nanocomposites displayed the highest specific surface area of 191 m^2^/g evidencing the synergistic effects between RGO and *h*-BN. Moreover, Co_3_O_4_/RGO/*h*-BN also displayed the highest specific capacity with stable reversibility on the high performance after 100 cycles and lower internal resistance. Interestingly, this novel nanocomposite exhibits outstanding high temperature performances with excellent cycling stability (100% capacity retention) and a decreased internal resistance at 150 °C.

## Introduction

Li-ion batteries energy storage devices are used as a power source for almost all electronic devices due to the superior benefits over other types of batteries^1–4^. However, the safety feature and the narrow temperature operating range of li-ion batteries remain a major obstacle for more complex applications of li-ion batteries such as in the oil industry, defense, automotive applications and aerospace that demand safe operation at wide temperature range (up to 150 °C). Li-ion batteries are known to operate effectively between −20 °C and 60 °C^5^. With the increasing demand for li-ion batteries, many research has been made on increasing its thermal stability and the upper operating temperature range.

When considering safety issues of li-ion batteries it is mainly related to thermal runaway. Conditions such as elevated temperature and high charge levels or overcharging abuses one or more of the battery components that results in what is called a short circuit leading to heat, fire or explosion. A process referred to as thermal runaway^6^. Thermal runaway mechanisms occur mainly at the electrodes and electrolytes. Thermal decomposition of the electrodes or electrolytes and reduction or oxidation of the electrolyte is the main cause of thermal runaways.

To solve this issue, many preventative measures have been investigated. Preventative measures can be the use of safety devices, that is, design devices that release high pressure and heat before thermal runaway but this is for engineers to set up new safe li-ion battery devices. However, what concerns scientists more is the inherent safety from electrodes, to electrolytes^7^. Compromising between the electrochemical performances and thermal stability is a challenge.

To overcome this issue, highly thermal stable two-dimensional (2D) materials that are based on hexagonal boron nitride (h-BN) can be very promising. This is because h-BN have high thermal stability even when compared to carbon materials^8^ such as graphene which can prevent thermal runaway events. Additionally, h-BN has equal strength to the already known strongest material which is graphene^9^. More importantly, h-BN is stable and inert against many chemicals including lithium^10^.

Due to the abovementioned properties, studies are ongoing on the use of 2D h-BN based anodes, for the safe operation of li-ion batteries. One such example is the use of 2D material as an artificial SEI layer to prevent lithium dendrite formation. Many research has been conducted on designing a stable SEI layer. However, the challenge is to design an SEI layer that has high electronic resistivity, high li-ion conductivity and permeability in addition to high mechanical strength^11^. Kai Yan *et al*. demonstrated a study that shows how graphene and h-BN can be used as SEI to prevent lithium dendrite formation. The study showed that the use of either graphene or h-BN on a lithium metal anode leads to a safer and stable cycle performance with alteration of SEI by the 2D nanomaterials^12^.

Despite the abovementioned advantages, some challenges remain when utilizing 2D materials as electrodes in energy storage applications. This includes the restacking when fabricated into electrodes which deteriorates 2D materials’ outstanding properties. The integration of 2D material with other 2D material can alleviate challenges related to restacking while benefiting from the properties of both^13,14^. In addition, the low electrical conductivity limits the electrochemical performance of h-BN^15^. The integration of conductive graphene to 2D layered h-BN could be a promising approach to improve the electrochemical performance of li-ion batteries. The addition of graphene can tune the electrical properties of h-BN^16^. Also, graphene’s electrical properties are greatly affected by the substrate^17^. Graphene on hexagonal boron nitride devices was found to exhibit the highest mobility reported on any substrate^18^. Moreover, combining the outstanding mechanical and chemical properties of h-BN with graphene can overcome the limitations of restacking and preserve the properties of both. The high mechanical strength of h-BN in synergy with graphene can accommodate metal oxide’s volume expansion when used in composite with transition metal oxides.

To this extent, Li *et al*. studied the synergistic effect of graphene and BN on the electrochemical properties of li-ion batteries. rGO/BN composite films with varying BN contents were prepared through vacuum filtration followed by annealing. Electrochemical studies showed that the use of graphene modified with 2 wt.% BN resulted in superior electrochemical performance achieving a reversible capacity of 197 mAh/g over 200 cycles, which is higher than rGO (127 mAh/g)^19^. However, the performance of the prepared rGO/BN composite was only studied at room temperature. Recently, the synergistic effect of graphene/h-BN in heat management of electronic devices have been highlighted in many studies by theoretically investigating the relaxation dynamics of photoexcited (PE) carriers^20–22^.

In this work, we report a novel composite made up of Reduced Graphene Oxide/hexagonal boron nitride based transition metal oxide (Co_3_O_4_/RGO/*h*-BN) nanocomposite for high temperature li-ion batteries. The Co_3_O_4_/RGO/*h*-BN nanocomposite was synthesized via MWI at low temperatures of 180 °C and without any additives such as surfactants and reducing agents etc. which shows the simplicity of the method. Owing to their high theoretical capacities, metal oxides such as Co_3_O_4_ were used in combination with RGO/h-BN. The as synthesized Co_3_O_4_/RGO/*h*-BN nanocomposites were used as anodes in li-ion batteries which showed enhanced electrochemical and thermal stability. Since batteries store energy electrochemically, the energy stored is proportional to the surface area of the active material and therefore the high surface area (191 m^2^/g) of the Co_3_O_4_/RGO/*h*-BN nanocomposites resulted in the highest specific capacity, long cycling stability, and a decreased internal resistance. Moreover, the thermal studies demonstrated that Co_3_O_4_/RGO/*h*-BN had the highest thermal stability up to 300 °C, which could prevent thermal runaway events and is reflected by the stable electrochemical performance (100% capacity retention and coulombic efficiency) at 150 °C for Co_3_O_4_/RGO/*h*-BN nanocomposites.

## Materials and Method

All the chemicals used in this investigation were AR-grade materials and used without further purification. Cobalt Acetate (II) tetrahydrate ((CH_3_COO)_2_Co.4H_2_O 95%), was purchased from Loba Chemie. Ethanol absolute (C_2_H_5_OH 99%), potassium permanganate (KMnO_4_) and hydrogen peroxide (H_2_O_2_ 30% Extra pure) were purchased from Scharlau. De-ionized water was used to prepare aqueous solutions. Hydrochloric acid (HCl > 37%) was purchased from Sigma Aldrich. Graphite fine powder extra pure (<50 μm, 99.5%) and sodium nitrate crystal extra pure (NaNO_3_) were purchased from Merck. On the other hand, Boron Nitride (BN) Micropowder (98% purity) was purchased from Graphene Supermarket.

### Graphene oxide (GO) synthesis

Graphene oxide was prepared using the modified hummer’s method. The synthetic procedure involved two processes: Oxidation of graphite to create graphite oxide followed by ultra-sonication to end up with graphene oxide. In a typical reaction, H_2_SO_4_ (115 ml) was stirred with graphite (2 g) for 15 min in an ice bath followed by slowly adding NaNO_3_ (2.5 g) and then KMnO_4_ (20 g) after 15 min later. The mixture was left under continuous stirring while in an ice bath for another 15 min. After 20 min, the mixture was heated at (32–40 °C) for 2 hrs with vigorous stirring. A color change from black to dark green was observed. Deionized water (230 ml) was then added to the above mixture while keeping the temperature below 50 °C in an ice bath. 20 min later, 30% H_2_O_2_ (20 ml) was added and a yellow color was observed. Finally, 10% HCl was added to the mixture followed by washing and centrifugation (HERAEUS - LABOFUGE 400 Centrifuge) until neutral pH. After these steps, the material was ultra-sonicated for 5 min and kept for drying at 60 °C.

### Functionalization of h-BN

h-BN was functionalized with OH groups or in other words hydroxylated to ease the attachment of metal oxides. In a typical reaction, 550 mg of h-BN was dissolved in 50 ml of distilled water followed by the addition of 30% H_2_O_2_ (10 ml) and H_2_SO_4_ (5 ml). The mixture was ultra-sonicated for 30 min followed by hydrothermal microwave irradiation (CEM One touch technology MARS 6) at 180 °C, 900 W and 150 psi for 15 min. The material was washed and centrifuged (HERAEUS - LABOFUGE 400 Centrifuge) and left for drying overnight at 60 °C.

### Synthesis of Co_3_O_4_ nanoparticles, Co_3_O_4_/RGO, and Co_3_O_4_/*h*-BN nanocomposites

To synthesis Co_3_O_4_, 0.2 M of cobalt (II) acetate tetrahydrate was dissolved in distilled water (30 ml) followed by stirring for 30 min. Then, the mixture was exposed to hydrothermal treatment under microwave irradiation (CEM One touch technology MARS 6) at 180 °C, 900 W and 150 psi for 59 min. For the synthesis of Co_3_O_4_/RGO, 50 mM of cobalt (II) acetate tetrahydrate Co (C_2_H_3_O_2_)_2_ was dissolved in 30 ml of graphene oxide (2 mg/ml) and then the same procedure was followed as for the pure Co_3_O_4_. Similarly, the same procedure as the Co_3_O_4_/RGO was followed to synthesis Co_3_O_4_/*h*-BN but in this case h-BN (2 mg/ml).

### Synthesis of Co_3_O_4_/RGO/*h*-BN nanocomposites

A 2:1 ratio of Co_3_O_4_/RGO to *h*-BN was used to synthesize the Co_3_O_4_/RGO/*h*-BN nanocomposites. Co_3_O_4_/RGO (60 mg) was dissolved in 30 ml of ethanol followed by 30 min stirring. Then, 15 ml of *h*-BN (2 mg/ml) was added to the solution. The mixture was then exposed to hydrothermal treatment under microwave irradiation (CEM One touch technology MARS 6) at 180 °C, 900 W and 150 psi for 59 min.

### Material characterization

Structural studies of the prepared nanocomposites were analyzed using X-ray diffraction (XRD) on a Rigaku MiniFlex600 X-ray diffractometer using Cu Kα radiation at a scanning rate of 2°/min. Chemical composition studies were performed on a Thermo Scientific Nicolet-iS10 Fourier transform infrared spectroscopy (FT-IR). Thermal gravimetric analysis (TGA) of the nanocomposites was recorded from ambient to 550 °C under N_2_ atmosphere at a heating rate of 5 °C/min on a STA7200 thermal analysis system. Differential Scanning Calorimeter (DSC) DSC7020 was equipped to measure the DSC thermal studies and was performed under N_2_ atmosphere at a heating rate of 10 °C/min from room temperature to 500 °C. Further spectral studies were performed for the obtained nanocomposites using an iRaman plus Raman spectroscopy. Morphological studies were conducted by Zeiss MERLIN Field Emission Scanning Electron Microscope (FE-SEM) field emission scanning electron microscopy and JEOL JEM-2100 F Transmission electron microscopy (TEM). Surface elemental analysis was performed using X-ray photoelectron spectroscopy (XPS) studies on ESCALABMK II X-ray photoelectron spectrometer with Mg-Ka radiation. The specific surface area was calculated using a Brunauer-Emmett-Teller (BET) method via N_2_ adsorption-desorption measurement on Micromeritics ASAP 2020. The pore size distribution was obtained by the Barrett–Joyner–Halenda (BJH) method.

### Electrochemical measurements

To study the electrochemical performances, 80 wt.% of the active material (nanocomposites), 10 wt.% of conductive agent-carbon black (Sigma-Aldrich) and 10 wt.% of binding agent-polyvinylidene fluoride (Sigma-Aldrich) was dissolved in 1:1 ethanol: dimethylsulfoxide (Sigma-Aldrich) solvent.  Homogenous slurry was obtained and was then coated on copper foil substrates and allowed to dry at 80 °C under vacuum. The working electrode was weighed which was then cut to form disks of 15 mm. The electrode was then assembled to CR2032 coin-type cell in an argon-filled glove box. Lithium metal was used as a counter electrode with polypropylene membrane separator (Celgard 2325) and 1 M LiPF_6_ in EC + DMC + DEC (1:1:1 vol.%) was used as the electrolyte. Galvanostatic charge/discharge curves were obtained using BST8–5A-CST eight-channel battery analyzer and Gamry 3000 electrochemical working station in a potential window of 0–3 V at different current densities. Gamry 3000 electrochemical working station was also used to measure cyclic voltammetry (CV) at a scan rate of 50 mV/s in a voltage range of 0–3 V and electrochemical impedance spectroscopy (EIS) by applying a perturbation voltage of 10 mV in the frequency range of 1 Hz and 100 kHz. Furthermore, testing at high temperature was performed via a bomb calorimeter vessel in which the positive and negative terminals of the battery were connected to the two electrodes of the vessel. The coin cell was put inside the vessel at the desired temperature for several hours to ensure that the temperature is stable. Electrochemical characterizations including galvanostatic charge/discharge, CV and EIS were then performed by connecting the bomb calorimeter vessel to the Gamry potentiostat.

## Results and Discussion

**Figure 1:**
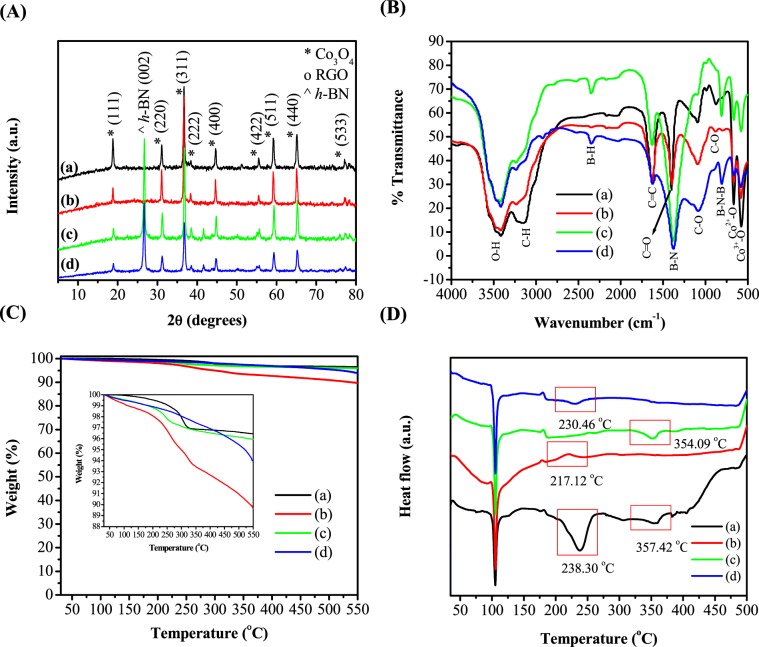
(**A**) XRD patterns, (**B**) FT-IR spectra, (**C**) TGA and (**D**) DSC curves of (a) Co_3_O_4_ nanoparticles, (b) Co_3_O_4_/RGO, (c) Co_3_O_4_/*h*-BN and (d) Co_3_O_4_/RGO/*h*-BN nanocomposites.

XRD studies were performed to investigate the phase structure of Co_3_O_4_, Co_3_O_4_/RGO, Co_3_O_4_/*h*-BN and Co_3_O_4_/RGO/*h*-BN as illustrated in Fig. 1(A). The diffraction peaks of Co_3_O_4_ nanoparticles can be indexed to the pure Co_3_O_4_ with normal cubic spinel structure (JCPDS card no. 76-1802). However, the increase in the peak intensity of (111) reveals that the Co_3_O_4_ nanoparticles are exposed of (111) planes, where the surface is mainly composed of Co^2+^ active sites. To prepare metal oxides like Co_3_O_4_, a basic medium is required, however, during our preparation of Co_3_O_4_ the only source of OH^−^ is from the hydrolysis of acetate anions. Thus, the H_2_O amount used was not sufficient for 0.2 M of cobalt acetate. This low amount of hydroxyl anions leads to the formation of Co_3_O_4_ nanocubes with (001) exposed planes^23,24^. Co_3_O_4_/RGO nanocomposites also show similar peaks of Co_3_O_4_ with normal cubic spinel structure. However, the XRD doesn’t show an obvious peak of RGO, it could be due to Co_3_O_4_ particles anchored on the surface of RGO and prevents exfoliated RGO sheets from face-to-face restacking following the reduction process. This confirms that there is an interconnection between Co_3_O_4_ and graphene. Furthermore, the low amount of RGO and low diffraction intensity of graphene compared to Co_3_O_4_, can cause the RGO peak to disappear^25,26^. For Co_3_O_4_/*h*-BN, the very well crystalline peak at 26° is attributed to the (002) diffraction peak of *h*-BN^27^. Furthermore, the XRD pattern of Co_3_O_4_/RGO/*h*-BN shows Co_3_O_4_ diffraction peak in addition to RGO and *h*-BN peaks. Co_3_O_4_ peaks of high crystallinity were formed. The (002) RGO peak in Co_3_O_4_/RGO/*h*-BN nanocomposites was also not very obvious with a broad peak due to the highly crystalline (002) *h*-BN peak indexed at 26°. It should be noted that for the XRD graphs of Co_3_O_4_/RGO, Co_3_O_4_/*h*-BN, and Co_3_O_4_/RGO/*h*-BN, the 111 plane matches the XRD of pure Co_3_O_4_ with normal cubic spinel structure. This could be due to the sufficient amount of hydroxyl anions in the solution during the preparation of Co_3_O_4_/RGO, Co_3_O_4_/*h*-BN, and Co_3_O_4_/RGO/*h*-BN as a lower concentration of cobalt acetate was used with the same amount of H_2_O used in Co_3_O_4_ synthesis^28^. Furthermore, from XRD, the d-spacing and the crystallite sizes were obtained as is shown in Table S1. The crystallite sizes of Co_3_O_4_ nanoparticles calculated using the Scherer equation is in the range of 20–45 nm. From the crystal sizes, it was observed that Co_3_O_4_/RGO have larger sizes of Co_3_O_4_ nanoparticles when compared to pure Co_3_O_4_ and Co_3_O_4_/*h*-BN this might be due to oxygen groups from RGO interact with Co_3_O_4_ and resulted in larger Co_3_O_4_ size, whereas the addition of RGO/*h*-BN reduced the size of Co_3_O_4_ nanoparticles. However, Co_3_O_4_ and Co_3_O_4_/*h*-BN showed similar Co_3_O_4_ particle sizes of 31 nm which shows no deviation of particle size distribution before and after incorporation of Co_3_O_4_ on *h*-BN sheets.

FT-IR was further used to characterize the chemical structure and to determine the surface functional groups and bonding nature of Co_3_O_4_, Co_3_O_4_/RGO, Co_3_O_4_/*h*-BN, and Co_3_O_4_/RGO/*h*-BN and as shown in Fig. 1(B). For Co_3_O_4_ and Co_3_O_4_/RGO, the two peaks at 581.38 cm^−1^ and 665.57 cm^−1^ are ascribed to the Co-O stretching vibration mode and the bridging vibration of O-Co-O bond, respectively suggesting the formation of a pure phase of Co_3_O_4_. The absorption bands at 1093.13 cm^−1^ and 1394.71 cm^−1^ are assigned to the C–O and C=O stretching vibrations. The C=C band at 1629.10 cm^−1^ is assigned to the bending of C=C aromatic rings of RGO. The existence of the C=C peak suggested that the sp^2^ structure of carbon atoms remained. However, the presence of the O–H band at 3411.07 cm^−1^ suggests the possibility of adsorbed water or hydroxyl (OH) group from GO as GO is considered to be hydrophilic. Furthermore, the FT-IR spectrum of Co_3_O_4_/*h*-BN shows characteristic peaks for hexagonal born nitride. The performed FT-IR spectral studies showed two strong absorption bands located at 1375 cm^−1^ and 813.24 cm^−1^ attributed to the in-plane B–N stretching vibrations and out-of-plane B–N–B bending vibrations of *h*-BN, respectively, while the band at 2530.47 cm^−1^ is assigned to the B-H bond^29^. In addition, the FT-IR spectrum shows peaks around 1629.71 cm^−1^ and 2794.52 cm^−1^, corresponding to C=O bonds and aliphatic C–H, respectively. Also, the peaks centered at 3416.09 cm^−1^ and 3239.38 cm^−1^ are attributed to the stretching modes of the H–N–H or O–H groups. Similarly, Co_3_O_4_/RGO/*h*-BN displayed a similar spectrum as Co_3_O_4_/*h*-BN. However, in the case of Co_3_O_4_/RGO/*h*-BN nanocomposites the peak centered at 1629.71 cm^−1^ is most likely due to the C=C bond of carbon atoms from RGO. From the above FT-IR result, it can be said that Co_3_O_4_, RGO, and *h*-BN based nanocomposites are achieved.

On the other hand, thermal studies were performed to test the thermal stability of the designed anode materials. The thermal stability and degradation behaviors of the Co_3_O_4_ nanoparticles, Co_3_O_4_/RGO and the BN based nanocomposites were investigated by TGA and the results are shown in Fig. 1(C) with the associated dW/dT curves displayed in Fig. S3. The TGA curve can be divided into three regions based on the weight losses, it was found that in the first region that is below 100 °C, only 1% decomposition is observed for the Co_3_O_4_/RGO nanocomposite that is due to the release of absorbed water. In the second region that is between 100 and 300 °C, Co_3_O_4_/RGO showed a 4% loss around 250 °C while Co_3_O_4_/*h*-BN showed a weight loss of only 2% around 225 °C. These losses are due to the decomposition of epoxy and hydroxyl groups found in RGO and hydroxyl groups in *h*-BN. This shows that the GO oxygen containing group were almost all reduced to RGO during the reduction process. After 250 °C, both Co_3_O_4_/RGO and Co_3_O_4_/*h*-BN display a gradual mass loss, which is due to the further removal of functional groups. At 550 °C, the TGA curves showed an overall weight loss of 10% for Co_3_O_4_/RGO and a weight loss of 3–6% for the *h*-BN based Co_3_O_4_ nanocomposites. On the other hand, Co_3_O_4_ showed only ~3% loss observed around 300 °C and remained stable up to 550 °C. For Co_3_O_4_/RGO/*h*-BN nanocomposites, a weight loss of only 0.5% was observed around 100 °C which is due to moisture. In the region between 100 °C and 300 °C, a weight loss of 1.5% was observed due to the reduction of oxygen containing functional groups from both RGO and *h*-BN. After 300 °C, a gradual decrease in mass loss was observed with a total weight loss of 6.5% at 550 °C due to the further removal of functional groups. It is noteworthy that the weight percentage of RGO in Co_3_O_4_/RGO nanocomposites was higher than in Co_3_O_4_/RGO/*h*-BN nanocomposites. Thus, Co_3_O_4_/RGO showed more weight loss than Co_3_O_4_/RGO/*h*-BN nanocomposites.

Correspondingly, the DSC curves of the Co_3_O_4_, Co_3_O_4_/RGO, Co_3_O_4_/*h*-BN, and Co_3_O_4_/RGO/*h*-BN nanocomposites are shown in Fig. 1(D). The DSC curve of Co_3_O_4_ nanoparticles has three major endothermic peaks. The first endothermic peak at around 105 °C is due to the evaporation of adsorbed water. The endothermic peak at 238.30 °C can be associated with the decomposition of the cobalt precursor. The third endothermic peak observed at 357.42 °C is attributed to the further decomposition of intercalated CH_3_COO– groups. The addition of RGO to Co_3_O_4_, converted the second endothermic peak to an exothermic peak at 217.12 °C, which may be due to the catalytic effect of RGO. Co_3_O_4_/*h*-BN only showed a third endothermic peak at 354.09 °C. Whereas, the addition of *h*-BN to Co_3_O_4_/RGO converted the second exothermic peak back to endothermic at 230.46 °C. For both Co_3_O_4_/RGO and Co_3_O_4_/RGO/*h*-BN nanocomposites, the third endothermic peak of Co_3_O_4_ disappears.

**Figure 2:**
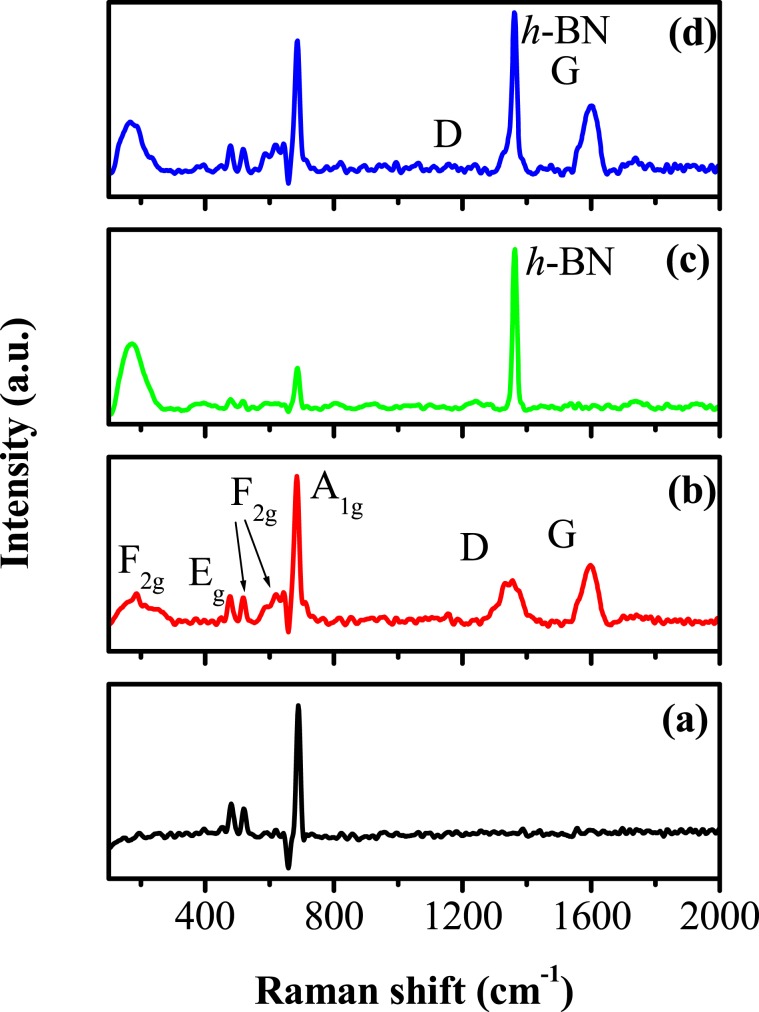
Raman spectra of (**a**) Co_3_O_4_ nanoparticles, (**b**) Co_3_O_4_/RGO, (**c**) Co_3_O_4_/*h*-BN and (**d**) Co_3_O_4_/RGO/*h*-BN nanocomposites.

To further investigate the chemical structure configuration, Raman spectra of Co_3_O_4_, Co_3_O_4_/RGO, Co_3_O_4_/*h*-BN, and Co_3_O_4_/RGO/*h*-BN were performed as illustrated in Fig. 2(a). The Raman spectrum of Co_3_O_4_ nanoparticles showed five Raman bands at around, 182 cm^−1^ (F_2g_), 478 cm^−1^ (E_g_), 521 cm^−1^ (F_2g_), 611 cm^−1^ (F_2g_) and 689 cm^−1^ (A_1g_) which confirms the spinel structure of Co_3_O_4_. The Raman mode at 689 cm^−1^ (A_1g_) corresponds to characteristics of the octahedral sites whereas 182 cm^−1^ (F_2g_), 478 cm^−1^ (E_g_), 521 cm^−1^ (F_2g_) and 611 cm^−1^ (F_2g_) are attributed to the combined vibrations of the tetrahedral site and octahedral oxygen motions^30^. For Co_3_O_4_/RGO nanocomposites, in addition to the five Co_3_O_4_ Raman bands, extra two clear peaks at 1327 cm^−1^ and 1584 cm^−1^ are detected, which corresponds to the D and G peaks of graphene^31,32^, respectively, as shown in Fig. 2(b). The D band is attributed to the breathing mode of k-point phonons of A_1g_ symmetry and the G bands to the E_2g_ phonon of sp^2^ C atoms. In addition, the D band results from the sp^3^ edge defects, disorder and loss of graphitic structure. These results are strong evidence that supports the existence of both RGO and Co_3_O_4_ in the prepared composites^33^. For Co_3_O_4_/*h*-BN, in addition to the five Co_3_O_4_ Raman active modes, a single peak located at 1365 cm^−1^ corresponds to an in-plane vibration (E_2g_) and is analogous to the well-known G peak in graphene which confirms the existence of hexagonal boron nitride as illustrated in Fig. 2(c). In the case of Co_3_O_4_/RGO/*h*-BN (Fig. 2(d)), an intense and sharp peak at 1365 cm^−1^ is observed in the spectrum which is close to the characteristic Raman peak of bulk h-BN materials. The spectra of Co_3_O_4_/RGO/*h*-BN exhibits two peaks at 1344 and 1589 cm^−1^, which indicates the presence of RGO sheets in the nanocomposites. However, D band of graphene is almost negligible indicating the lower number of defects in the graphene structure^34,35^.

**Figure 3:**
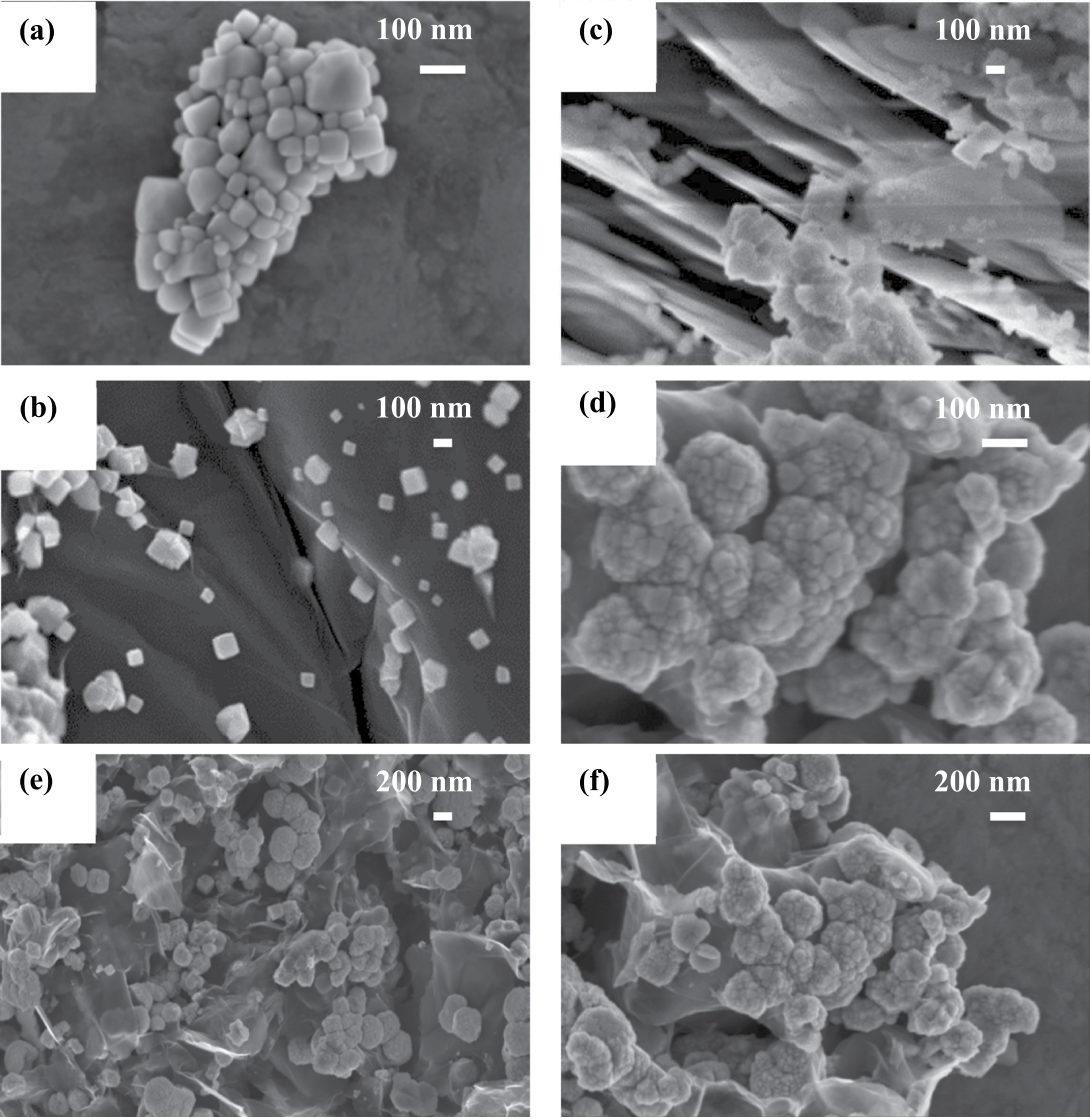
FE-SEM (Field Emission Scanning Electron Microscope) images of (**a**) Co_3_O_4_ nanoparticles, (**b**) Co_3_O_4_/RGO, (**c**) Co_3_O_4_/*h*-BN and (**d**–**f**) Co_3_O_4_/RGO/*h*-BN.

FE-SEM was employed to investigate the morphologies of Co_3_O_4_ nanoparticles, Co_3_O_4_/RGO, Co_3_O_4_/*h*-BN, and Co_3_O_4_/RGO/*h*-BN nanocomposites. Pure Co_3_O_4_ nanoparticles showed cubic like structure as shown in Fig. 3(a). FE-SEM images for Co_3_O_4_/RGO nanocomposites show Co_3_O_4_ nanoparticles residing on the reduced graphene oxide sheets with homogenous dispersion as shown in Fig. 3(b). Similarly, for the Co_3_O_4_/*h*-BN nanocomposites, cube like Co_3_O_4_ nanoparticles residing on the surface of h-BN sheets was observed as displayed in Fig. 3(c). On the other hand, cubic nanoparticles incorporated in transparent h-BN sheets and RGO sheets were observed for the Co_3_O_4_/RGO/*h*-BN nanocomposites as shown in Fig. 3(d–f). FE-SEM further confirmed the successful synthesis of Co_3_O_4_ nanoparticles, Co_3_O_4_/RGO, Co_3_O_4_/*h*-BN, and Co_3_O_4_/RGO/*h*-BN nanocomposites.

**Figure 4:**
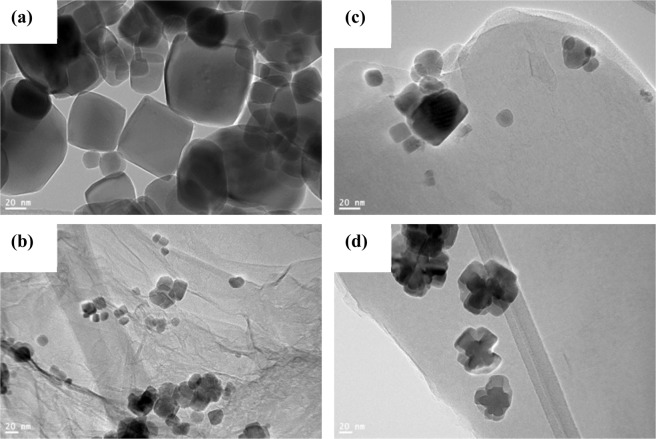
HR-TEM images of (**a**) Co_3_O_4_ nanoparticles, (**b**) Co_3_O_4_/RGO, (**c**) Co_3_O_4_/*h*-BN and (**d**) Co_3_O_4_/RGO/*h*-BN.

More detailed structures of the prepared nanocomposites were observed using high resolution TEM (HR-TEM). The presence of Co_3_O_4_ incorporated onto the graphene sheets and *h*-BN sheets is seen. In addition, to morphology, the d-spacing of the nanostructures can also be obtained using HR-TEM. The higher resolution TEM of pure Co_3_O_4_ nanoparticles revealed that it is cubic as shown in Fig. 4(a) which is consistent with the FE-SEM result and further supports the cubic morphology. Furthermore, HR-TEM images were used to indicate that the RGO and BNNS consisted of a few atomic layers as shown in Fig. 4(b–d). Structural studies using XRD and morphological studies using FE-SEM and HR-TEM support each other and confirm the successful preparation of Co_3_O_4_/RGO/*h*-BN nanocomposites that consists of highly ordered and crystalline cubic structure incorporated into the conductive RGO matrix with a thin layer of thermally stable h-BN sheets. This unique structural features could help promote smooth channels for ion diffusion and alleviate the volume expansion that could occur during the charge and discharge process and inhibit the deterioration of the stable crystal structure^36^.

**Figure 5:**
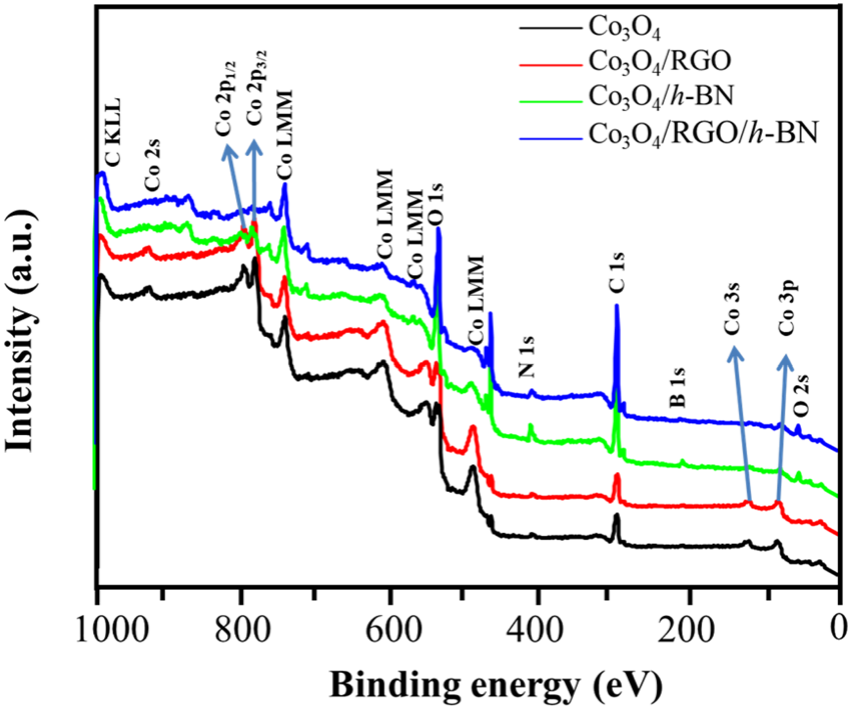
XPS spectra of Co_3_O_4_ nanoparticles, Co_3_O_4_/RGO, Co_3_O_4_/*h*-BN and Co_3_O_4_/RGO/*h*-BN.

XPS was also performed to study the surface elemental composition of the prepared nanocomposites. The XPS spectra of Co_3_O_4_, Co_3_O_4_, Co_3_O_4_/*h*-BN, and Co_3_O_4_/RGO/*h*-BN are shown in Fig. 5. Two major peaks corresponding to Co 2p_3/2_ (785.18 eV) and Co 2p_1/2_ (760.49 eV) are observed which corresponds to a typical Co_3_O_4_ phase with both Co^2+^ and Co^3+^ cations^37^. Those two peaks decrease in intensity and almost disappear in Co_3_O_4_/RGO/*h*-BN which is most likely because in Co_3_O_4_/RGO/*h*-BN the Co_3_O_4_ surface is coated with thin sheets of RGO as shown in the FE-SEM images. The high resolution C 1 s peak of Co_3_O_4_/RGO/*h*-BN in Fig. S3(a) could be deconvoluted into four peaks corresponding to the C–C or C=C (285.32 eV), C–O (286.85 eV), C=O (287.38 eV) and O–C=O (288.84 eV) of graphene oxide. It can be seen that the oxygen containing functional groups have low intensity, further confirming the successful reduction of graphene oxide^38^. The high content of carbon in the surface of Co_3_O_4_/RGO/*h*-BN nanocomposites, when compared to Co_3_O_4_/RGO is a further confirmation of the coating of the Co_3_O_4_ with RGO sheets in Co_3_O_4_/RGO/*h*-BN nanocomposites. The appearance of a carbon peak in Co_3_O_4_/*h*-BN could be due to adventitious graphitic carbon on the surface and the adsorbed carbon dioxide. The N 1 s peak at 402 eV and the N 1 s peak at 198.76 eV in Co_3_O_4_/RGO/*h*-BN nanocomposites correspond to the B-N bonds however they both have low signal intensities due to the low surface density of *h*-BN in Co_3_O_4_/RGO/*h*-BN nanocomposites. The contents of both boron and nitrogen peak in Co_3_O_4_/RGO/*h*-BN nanocomposites decrease when compared to Co_3_O_4_/*h*-BN, suggesting the high carbon content in the surface of Co_3_O_4_/RGO/*h*-BN^39,40^. The high resolution O 1 s peak of Co_3_O_4_/RGO/*h*-BN nanocomposites in Fig. S3(b) can be deconvoluted to two peaks at 530.4 eV and 533.15 eV which is attributed to oxygen lattice atoms and adsorbed water, respectively^41^.

The specific surface areas were measured using the BET method as shown in Fig. S2 and summarized in Table S1. The specific surface area of pure Co_3_O_4_ is 41 m^2^/g. Co_3_O_4_/RGO nanocomposites resulted in a higher BET surface area of 54 m^2^/g which could be due to the incorporation of graphene sheets into Co_3_O_4_. On the other hand, the measured BET surface area of Co_3_O_4_/*h*-BN nanocomposites resulted in a lower surface area of 12 m^2^/g. Interestingly, Co_3_O_4_/RGO/*h*-BN nanocomposites displayed the highest BET surface area of 191 m^2^/g, respectively. Furthermore, BJH calculations show that the pore size distribution of Co_3_O_4_ nanoparticles and Co_3_O_4_/RGO, Co_3_O_4_/*h*-BN and Co_3_O_4_/RGO/*h*-BN nanocomposites is in the range of 16, 10, 47 and 40 nm, respectively. The large specific surface area of Co_3_O_4_/RGO/*h*-BN nanocomposites is due to the unique structure of the composite made up of crystalline Co_3_O_4_ cubes, the synergistic effect of RGO and *h*-BN in addition to the well exfoliated sheets of both RGO and *h*-BN. The larger specific surface area of Co_3_O_4_/RGO/*h*-BN make them good candidates as anode materials due to the high lithium ions absorption capability by increasing the active sites and providing good contact between the electrode material and electrolyte^42^.

**Figure 6:**
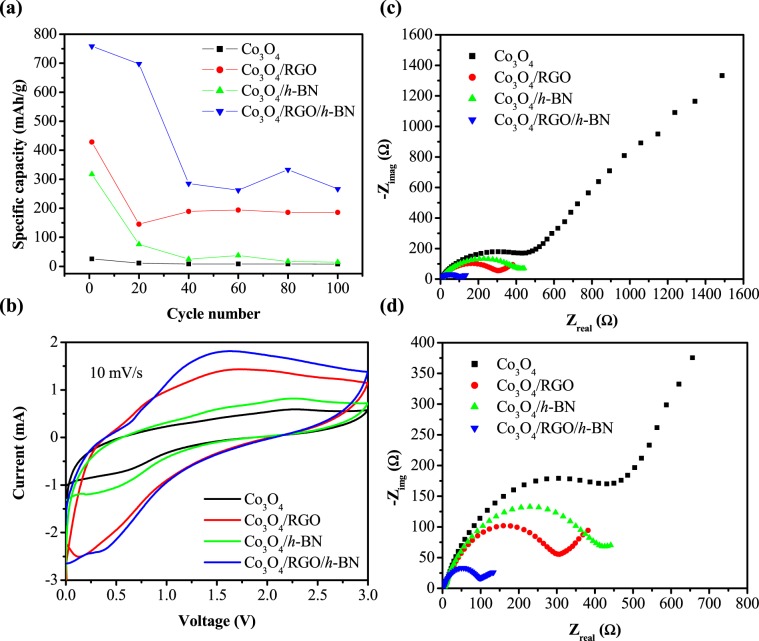
(**a**) Cyclic performance of Co_3_O_4_ (0.5–2.5 V at a current density of 62.5 mA/g), Co_3_O_4_/RGO (0.5–2.7 V at a current density of 156 mA/g), Co_3_O_4_/*h*-BN (0.5–2.2 V at a current density of 62.5 mA/g) and Co_3_O_4_/RGO/*h*-BN (0.5–2.7 V at a current density of 208 mA/g) for the 1^st^, 20^th^, 40^th^, 60^th^, 80^th^ and 100^th^ cycle. (**b**) Cyclic voltammetric curve and (**c**,**d**) EIS spectra of Co_3_O_4_, Co_3_O_4_/RGO, Co_3_O_4_/*h*-BN and Co_3_O_4_/RGO/*h*-BN performed after 100 charge/discharge cycles.

To test the electrochemical performance, all the four Co_3_O_4_ based composites were galvanostatically charged and discharged for 100 cycles. Figure 6(a) shows the cyclic performance of Co_3_O_4_, Co_3_O_4_/RGO Co_3_O_4_/*h*-BN, and Co_3_O_4_/RGO/*h*-BN nanocomposites for the 1^st^, 20^th^, 40^th^, 60^th^, 80^th^ and 100^th^ cycles. As expected, Co_3_O_4_/RGO/*h*-BN displayed the highest specific capacities with an initial charge capacity of 758.33 mAh/g, followed by Co_3_O_4_/RGO with a charge capacity of 428.12 mAh/g with reversible capacities of 266.6 mAh/g and 185.93 mAh/g after 100 cycles for Co_3_O_4_/RGO and Co_3_O_4_/RGO/*h*-BN nanocomposites, respectively. Co_3_O_4_ displayed very low initial specific capacities of only 25.5 mAh/g and a reversible capacity of only 8 mAh/g. Interestingly, Co_3_O_4_/*h*-BN also displayed high specific capacity initially of 317 mAh/g which decreased dramatically to 14.5 mAh/g after the 100^th^ cycle which is slightly higher than Co_3_O_4_ which shows the potential of *h*-BN in increasing the performance of Co_3_O_4_ in terms of specific capacity although Co_3_O_4_/*h*-BN displayed the lowest specific surface area. Certain modifications on the preparation of Co_3_O_4_/*h*-BN nanocomposites and understanding the chemistry behind the two in terms of interaction and so forth can make them potential candidates in li-ion battery energy storage devices. CV of the pure Co_3_O_4_, graphene based Co_3_O_4_ nanocomposites, *h*-BN based Co_3_O_4_ nanocomposites, and Co_3_O_4_/RGO/*h*-BN were performed in a potential voltage window between 0 and 3.0 V at a scan rate of 10 mV/s to further evaluate the electrochemical properties as illustrated in Fig. 6(b). A cathodic peak appeared at about 0.52 V for all the nanocomposites which is a result of the reduction of Co_3_O_4_ to Co metal, formation of clusters between Co and Li_2_O and formation of the solid electrolyte interphase (SEI) layer on the active materials^43^. As for the oxidation or the anodic scan, two peaks one at 2.25 V after the first cycle was observed which resulted from the reversible oxidation of metal Co to Co_3_O_4_^44,45^. The oxidation and reduction peaks are also attributed to the insertion/extraction of lithium into/from graphene. The overlap of the second and the third cycle is an indication of an enhanced cycling performance^46^. Interestingly, CV results are in line with the galvanostatic charge/discharge studies as Co_3_O_4_/RGO/*h*-BN displayed the highest current. EIS was also plotted for all the Co_3_O_4_ based nanocomposites performed after 100 charge-discharge cycles as shown in Fig. 6(c,d). From the Nyquist plots, it was observed that there is a depressed semicircle in the high-frequency region because of the resistance caused by the SEI layer (R_SEI_). In the medium frequency region, a broad arc is observed which is attributed to the Li^+^ charge-transfer resistance (Rct) on the electrode/electrolyte interface and an inclined line in the low frequency because of Warburg resistance (W). A lower resistance curve was observed for the Co_3_O_4_/RGO/*h*-BN nanocomposite when compared to the other nanocomposites. The smaller semicircle in the high-medium frequency region of Co_3_O_4_/RGO/*h*-BN nanocomposites is an indication of a faster charge-transfer and smaller internal electrochemical resistance while at the low frequency region, the steeper tail of Co_3_O_4_/RGO/*h*-BN nanocomposites means lower ion diffusion resistance and enhanced mass transport^47,48^ thus an enhanced electrochemical performance which is in agreement with the previous electrochemical studies.

**Figure 7:**
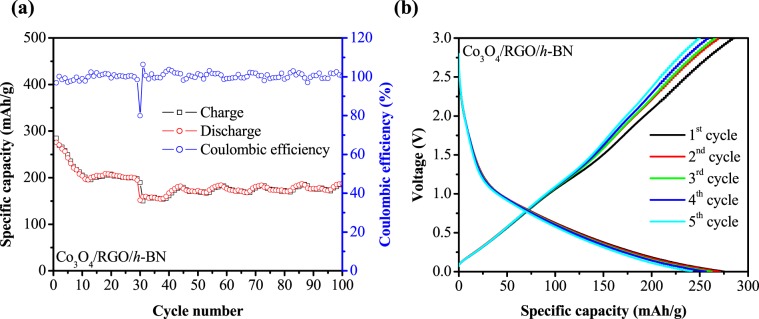
(**a**) Cyclic performance (0–3.5 V at a current density of 208 mA/g) and (**b**) galvanostatic charge/discharge curves (0–3.5 V at a current density of 208 mA/g) for the first 5 cycles of Co_3_O_4_/RGO/*h*-BN nanocomposites.

Thus, to further evaluate their electrochemical studies of the  Co_3_O_4_/RGO/*h*-BN nanocomposites, cyclic performance was conducted for another 100 charge/discharge cycles as shown Fig. 7(a) and galvanostatic charge/discharge curves for the first 5 cycles in Fig. 7(b). Cycling performance showed that the coulombic efficiency for the discharge/charge curve of Co_3_O_4_/RGO/*h*-BN nanocomposites was 100% for another 100 cycles with 186.58/187.40 mAh/g charge/discharge capacities after a total of 200 cycles. These results suggest that the as prepared Co_3_O_4_/RGO/*h*-BN can retain its initial morphology with stable reversibility on the high performance after 200 cycles. The galvanostatic charge/discharge of Co_3_O_4_/RGO/*h*-BN nanocomposites for the 1^st^ 5 cycles displayed in Fig. 7(b) shows the initial charge/discharge capacities of 284.80/275.90 mAh/g and minor irreversible capacities losses from the 2^nd^ to the 5^th^ cycle which is estimated to be only 8%.

The high electrochemical performance of Co_3_O_4_/RGO/*h*-BN nanocomposites is due to the synergistic effect of Co_3_O_4_, RGO, and h-BN. The abundant electrochemical active sites of cubic Co_3_O_4_ can help adsorb more li-ions and shorten the electron/ions transfer path. RGO could act as a conductive carbon matrix and thus facilitate electron transfer. Whereas, the introduction of the thermally stable 2D h-BN could help maintain the structure of RGO by preventing it’s restacking. Moreover, it is reported that the addition of heteroatom (e.g., S, N, and P) to a carbon matrix could help for rapid ion insertion/de-insertion and fast electron transportation^49,50^.

The electrochemical performance of Co_3_O_4_/RGO/*h*-BN was then studied at a higher operating temperature of 150 °C. If we have a closer look at the thermal studies conducted mainly the TGA and dW/dT curves shown in Fig. 1(C) and Fig. S1 it is apparent that Co_3_O_4_/RGO/*h*-BN nanocomposite was the most thermal stable. In addition, thermal studies for the nanocomposites were also performed when they were formed into a composite with activated carbon and PVDF as shown in Fig. S4. From the curves, no degradation was observed until 150 °C while degradation peaks appeared at around 160 °C which is due to PVDF thus further confirming the thermal stability of the prepared nanocomposites and have the tendency to operate well at 150 °C.

**Figure 8:**
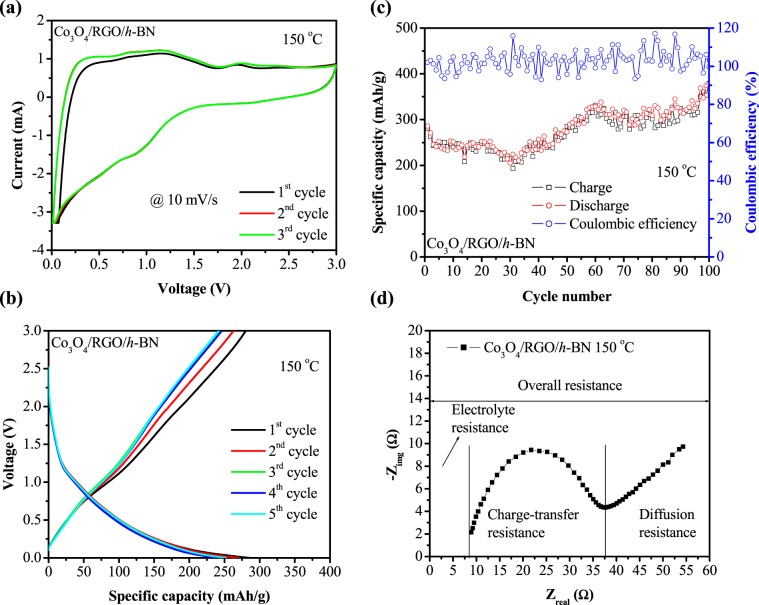
(**a**) Cyclic voltammetric (CV), (**b**) galvanostatic charge/discharge curves (0–3.0 V at a current density of 1250 mA/g), (**c**) cycling performance (0–3.0 V at a current density of 1250 mA/g) and (**d**) EIS spectra of Co_3_O_4_/RGO/*h*-BN nanocomposites performed at 150 °C.

Figure 8(a) shows the CV curve of Co_3_O_4_/RGO/*h*-BN nanocomposites at 150 °C performed in a potential voltage window between 0 and 3.0 V scanned at a rate of 10 mV/s for 3 cycles. The CV curve of Co_3_O_4_/RGO/*h*-BN nanocomposites at 150 °C exhibited an ideal CV curve of Co_3_O_4_ based anodes. However, it was observed that the current response of Co_3_O_4_/RGO/*h*-BN nanocomposites increased with stronger and sharper peaks, indicating the role of high temperature in enhancing the current-voltage performance of Co_3_O_4_/RGO/*h*-BN nanocomposites. Galvanostatic charge/discharge curves of Co_3_O_4_/RGO/*h*-BN nanocomposites at 150 °C for 5 and 100 cycles at a current density of 1250 mA/g are shown in Fig. 8(b,c). It’s noteworthy that a higher current density is required at high temperatures. An increase in the specific capacity was observed with cycle when the operating temperature was increased to 150 °C. From Fig. 8(c) it can be observed that an initial charge/discharge capacity of 280.21/286.04 mAh/g was obtained followed by a steady and gradual increase in capacity in the subsequent cycles. This increase upon cycling has been previously reported for carbon based metal oxides and could be due to several reasons such as electrochemical reversibility of the lithium oxide formation/decomposition and electrolyte reactions. Thus, a coulombic efficiency of higher than 100% was observed due to the reversible insertion of Li_2_O into the metal particles. This shows that high temperatures effectively promote decomposition of lithium oxide, electrolyte reactions, and the electrode material can be constantly activated which improves reaction kinetics with increased interfacial compatibility between the electrolyte and the electrode material^51–54^. Another contributing factor to the increase of capacity upon cycling could be due to the change in morphology of the composite with the increase of cycling number. The morphology of the composite would change to result in a structure that has a higher volume and surface area. This structural change increases the ability of the electrode material to adsorb and store a larger amount of Li^+^ ions^55^. Recently, this phenomenon (the increase in capacity upon cycling) was also observed in sodium-ion batteries and was due to co-intercalation reaction where Na atoms that are aggregated and stored at a specific location inside a carbon material during discharge are released, creating a space for more Na atoms in the next charging process, consequently increasing the capacity with cycling and resulting in a coulombic efficiency greater than 100%^56^. Moreover, the superior li-ion performance in terms of specific capacity observed at higher temperatures when compared to room temperature could be also explained through Arrhenius equation, that is, since li-ion batteries undergo a chemical reaction, temperature increases the rate of the reaction which increases the chemical reaction leading to an increase in the specific capacity. Kilibarda, G. *et al*. described other two factors that could also influence the increase in specific capacity as the temperature increases which includes the electrical/ionic conductivity of the electrolyte, increases with increasing temperature due to the low viscosity at high temperatures which increases the specific capacity. The other factor could be due to the drop in the ohmic potential or the decrease in the internal resistance of the cell which is also confirmed by the EIS plot performed at 150 °C as shown in Fig. 6(d). The increase of temperature resulted in a decrease in the potential drop, which causes a potential increase of the cell and thus increasing the specific capacity^57^. Furthermore, in contrast to the EIS conducted at room temperature, at high temperatures an additional resistance around 8 ohms is experienced which rises from the electrolyte.

## Conclusions

We have developed a novel Co_3_O_4_/RGO/*h*-BN nanocomposite using a facile MWI route as anodes for li-ion batteries. The prepared nanocomposites displayed high thermal stability making them good candidates in sustaining li-ion batteries thermal runaway. BET surface area showed that Co_3_O_4_/RGO/*h*-BN nanocomposites showed the highest specific surface area (191 m^2^/g) which is due to the high surface area of the well exfoliated graphene and *h*-BN sheets in addition to the synergistic effect between the two. Based on the advantages of their highest thermal stability and specific surface area, the Co_3_O_4_/RGO/*h*-BN anodes had the highest electrochemical response in terms of charge/discharge capacity, cyclic performance (100% capacity retention) and 100% coulombic efficiency even at high temperatures of 150 °C for 100 cycles. No short circuits, thermal runaway events or capacity decay was observed which makes Co_3_O_4_/RGO/*h*-BN nanocomposites potential candidates to be utilized as safe energy storage devices and especially in applications that operate at high temperatures. Specifically, can be utilized in industry to measure the ‘downhole’ pressure in oil wells and other oil applications where high temperatures are encountered ranging from 80 to 200 °C. In such high temperature environments, where batteries need to have an overall good electrochemical performance and should have the ability to operate safely over the wide range of high temperatures.

## Supplementary information

Supplementary Information

## Data Availability

All data generated or analysed during this study are included in this published article (and its Supplementary Information files).
