# Supplementary material for: Two dimensional (2D) reduced graphene oxide (RGO)/hexagonal boron nitride (h-BN) based nanocomposites as anodes for high temperature rechargeable lithium-ion batteries
Source: Sci Rep. 2020 Feb 5;10:1882. doi: 10.1038/s41598-020-58439-z (PMC7002573; doi:10.1038/s41598-020-58439-z)
Supplement: Supplementary file 1 — Supplementary Information [file 41598_2020_58439_MOESM1_ESM.docx]

**Supplementary information:**

**Two dimensional (2D) reduced graphene oxide (RGO)/hexagonal boron nitride (*h*-BN) based nanocomposites as anodes for high temperature rechargeable lithium-ion batteries**

Yasmin Mussa^1^, Faheem Ahmed^1^, Muhammad Arsalan^2^, Edreese Alsharaeh*^1^

^1^College of Science and General Studies, Alfaisal University, P.O. Box 50927, Riyadh, 11533, Saudi Arabia

^2^EXPEC Advanced Research Center, Saudi Aramco, P.O. Box 5000, Dhahran, 31311, Saudi Arabia

*[ealsharaeh@alfaisal.edu](mailto:ealsharaeh@alfaisal.edu)

Figure S1: dW/dT curves of (a) Co_3_O_4_ nanoparticles, (b) Co_3_O_4_/RGO, (c) Co_3_O_4_/*h*-BN and (d) Co_3_O_4_/RGO/*h*-BN nanocomposites.

Figure S2: Nitrogen adsorption/desorption isotherms of Co_3_O_4_ nanoparticles, Co_3_O_4_/RGO, Co_3_O_4_/*h*-BN and Co_3_O_4_/RGO/*h*-BN nanocomposites.

**(a)**

**(b)**

Figure S3: XPS characterizations of Co_3_O_4_/RGO/*h*-BN nanocomposites. (a) C 1s and (b) O 1s peaks.

The degradation of the Co_3_O_4_ nanoparticles started at over 300 °C, and for Co_3_O_4_/RGO two degradation temperatures were observed at over 250 °C and 325 °C. However, for Co_3_O_4_/*h*-BN the degradation occurred at around 240 °C. In case of Co_3_O_4_/RGO/*h*-BN nanocomposites, no clear degradation was observed until 350 °C which showed better thermal stability. This result demonstrates that the thermal stability of the Co_3_O_4_/RGO/*h-*BN nanocomposites is improved because of the synergy effect of RGO and *h*-BN.

Figure S4: DSC plots of (a) Co_3_O_4_ nanoparticles, (b) Co_3_O_4_/RGO, (c) Co_3_O_4_/*h*-BN and (d) Co_3_O_4_/RGO/*h*-BN nanocomposites when formed into composites with activated carbon and PVDF.

Table S1: Summarized d-spacing and sizes from of Co_3_O_4_ from XRD.

| Sample | 2-theta (deg) | | d (ang.) | FWHM (deg) | Size (nm) | BET specific surface area (m^2^/g) |
| --- | --- | --- | --- | --- | --- | --- |
| Co_3_O_4_ | Co_3_O_4_ | 36.68 | 2.45 | 0.285 | 30.7 | 41 |
| Co_3_O_4_/RGO | Co_3_O_4_ | 36.63 | 2.88 | 0.20 | 41.9 | 54 |
| Co_3_O_4_/*h*-BN | Co_3_O_4_ | 36.80 | 2.44 | 0.283 | 30.9 | 12 |
| Co_3_O_4_/RGO/*h*-BN | Co_3_O_4_ | 36.77 | 2.44 | 0.417 | 20.9 | 191 |
